# Pipeline for previously stented basilar trunk aneurysm: a case focusing on how the pipeline should be deployed

**DOI:** 10.1186/s41016-018-0134-7

**Published:** 2018-11-02

**Authors:** Fei Liang, Yupeng Zhang, Yuntao Di, Feng Guo, Chuhan Jiang

**Affiliations:** 1 Department of Interventional Neuroradiology, Beijing Neurosurgical Institute and Beijing Tiantan Hospital, Capital Medical University, Beijing, 100050 China; 2 Department of Neurosurgery, The People’s Hospital of Tangxian County, Tangxian, Hebei China

**Keywords:** Previously stented aneurysm, Basilar artery, Pipeline embolization device

## Abstract

**Background:**

Recurrent, previously stented basilar trunk aneurysms pose significant challenges to either microsurgical clipping or traditional endovascular treatment. We here presented an intriguing case that was successfully treated by the pipeline embolization device (PED; ev3/Covidien Neurovascular, Irvine, California, USA).

**Case presentation:**

A 62-year-old male found a recurrent aneurysm, which had been treated with coiling and stent-assisted coiling before. The patient came to our center seeking for a third treatment. In our center, a single PED and additional coils were used to treat this refractory aneurysm. The whole length of the PED was delicately implanted in the previous Enterprise stent (Cordis Corporation, Miami FL, USA), which resembled the double-layer flow diverter FRED (Microvention, Tustin, California, USA). The inflow zone of the aneurysmal sac was further coiled considering that this aneurysm had ruptured. No procedure-related complications occurred. Follow-up angiogram at 6 months indicated that the intractable aneurysm was completely occluded and the patient was free from any neurologic deficit.

**Conclusions:**

This is a case that adds knowledge to improve the poor performance of flow diverters in previously stented aneurysms. However, future studies with larger group of patients are needed to further test the safety and efficacy of this technique.

## Background

Ruptured aneurysms of the basilar arteries have a high mortality and morbidity rate and therefore require an instant procedure to prevent them from rebleeding. These aneurysms remain a therapeutic challenge considering that the basilar trunk is a perforator-rich area and deeply located in the intracranial space. Both surgical clipping and standard endovascular coil embolization would predispose the patients at great risk of post procedural complications [1–3]. Traditional endovascular techniques like coiling and SAC have become the first-line treatment of these aneurysms in that the safety and effectiveness of these two procedures are acceptable. However, to apply a technique which failed twice in this case is not rational.

PED is designed to obliterate the aneurysm by promoting thrombosis within the aneurysm and neointimal growth across the neck [4]. It has been successfully used in the treatment of large and giant wide-necked aneurysms in cavernous segment of ICA [5], but the use of PED to manage a previously stented basilar trunk aneurysm has rarely been reported. On the one side, the low porosity of PED might bring about severe brain stem ischemia. On the other side, the efficacy of implanting PED in a pre-existing stent has been proved unfavorable [6]. Here, we present the first successful treatment of a refractory and ruptured basilar trunk aneurysm with our unique way of deploying PED in an Enterprise stent, which we think may help to improve occlusion rate and lowering post procedural ischemia rate in this critical setting.

## Case presentation

**Fig. 1:**
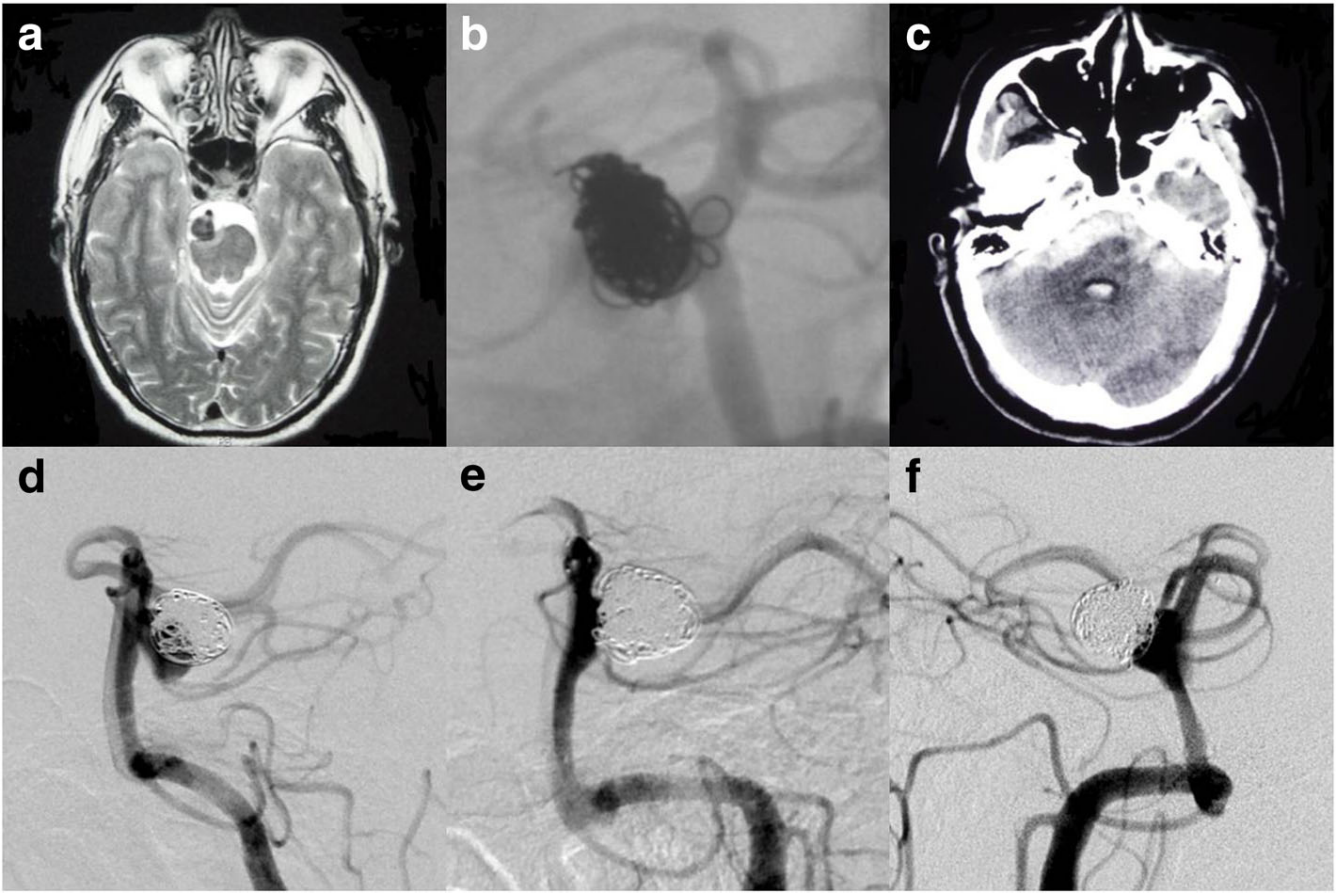
**a** The MRI indicated an aneurysm in front of the middle segment of the basilar artery. **b** Control DSA indicated near complete occlusion of the aneurysm. Ten years later, the patient presented with SAH suddenly (**c**) and DSA confirmed recurrence of the aneurysm (**d**). **e** The aneurysm was then treated again with robust packing of coils and an Enterprise stent. **f** Despite all these efforts, the coils were compacted and inflow zone was again patent as revealed by DSA

An unruptured basilar artery aneurysm was found due to a history of headaches in a 52-year-old male (Fig. 1a). The aneurysm was treated with simple coiling. Control angiography indicated near complete occlusion of the aneurysm, and the patient was free from any neurological deficit (Fig. 1b). Ten years later, the patient suddenly presented with subarachnoid hemorrhage (SAH) (WFNS grade 5) (Fig. 1c) and the digital subtraction angiography (DSA) confirmed recurrence of the aneurysm (Fig. 1d). After excluding the need to place an external ventricular drainage (EVD), the ruptured aneurysm was then treated by SAC with an Enterprise sized 4.5 mm × 22 mm 3 days after the ictus of SAH (Fig. 1e). This patient experienced right hemiparesis post procedure, possibly due to cerebral vasospasm, robust packing of coils, and the perforators covered by the stent. Then, we put the patient on rehabilitation and the patient recovered well (mRS = 1). Follow-up DSA 9 months later manifested with persist filling of contrast in the inferior portion of the aneurysm (Fig. 1f).

**Fig. 2:**
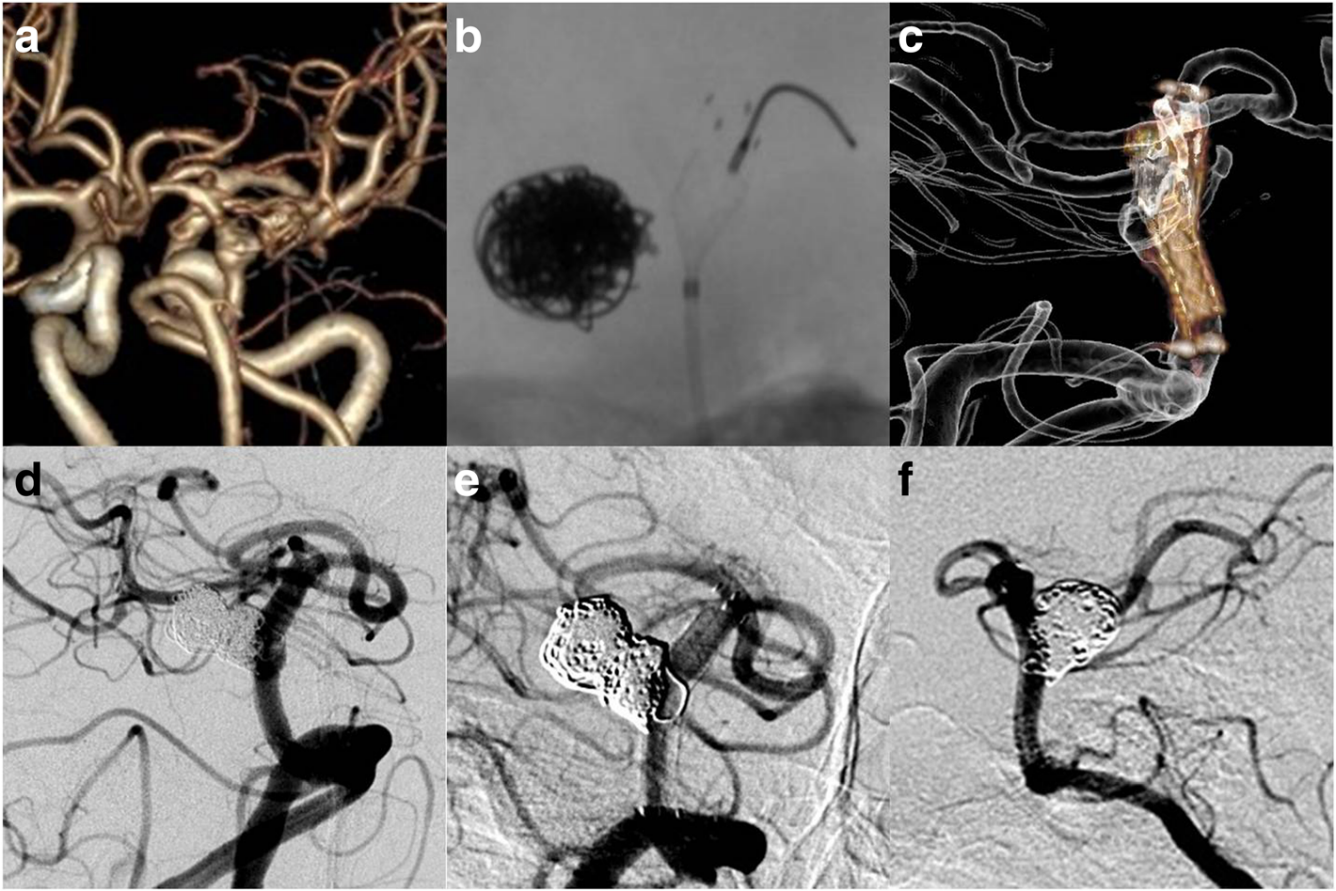
**a** CTA on admission suggested the recurrence of the aneurysm with fusiform morphology. **b** The distal end of the PED was deployed proximal to the distal end of the previous Enterprise stent. **c** Dyna-CT indicated that the entire PED was deployed within the Enterprise. **d** Adjunctive coiling was performed to secure the aneurysm. Right anterior oblique (**e**) and lateral view (**f**) of DSA indicated complete occlusion of the aneurysm on follow-up 6 months later

The patient came to our center seeking for a third treatment. CTA on admission indicated that the aneurysm was fusiform in morphology, possibly a dissecting aneurysm (Fig. 2a). Based on perceived high risk of rebleeding (according to the irregular shape and history of rupture) and recurrent nature of this aneurysm, we decided on consensus in the interdisciplinary discussion to treat this aneurysm with a single PED and adjunctive coiling. The patient was premedicated with a daily dose of 100 mg aspirin and 75 mg clopidogrel for 5 days. Thromboelastogram (TEG) suggested adequate response to the clopidogrel (inhibition rate 32.8%), so the procedure was performed under general anesthesia with systemic heparinization. Right femoral artery was canalized with an 8F arterial sheath, and a tri-axial system was then constructed with a 8F guiding catheter (Codman, Raynham, Massachusetts, USA), 6 F Navien (ev3/Covidien, Mansfield, Massachusetts, USA), and a Marksman microcatheter (EV3, Irvine, CA, USA). The Marksman was positioned beyond the P2 segment of the left posterior cerebral artery through the Navien. In our center, we used to open the PED distal to the landing zone, like in the PCA or in the distal part of basilar artery, then we would retract the half-opened PED to the target place. However, for this aneurysm with a stent in parent artery, we deployed a 4 × 16-mm sized PED in situ and made sure that the distal end of PED was proximal to the strut of the Enterprise stent so that the PED would not be stuck at the struts of Enterprise (Fig. 2b). And the proximal end of PED should be distal to the proximal end of the previous stent. As a result, the whole length of PED was within the Enterprise (Fig. 2c) and worked as an inner layer while the Enterprise acted as a scaffold. This complex resembled the double-layer flow diverter FRED. Due to that this aneurysm once ruptured, we further coiled the aneurysm through a pre-jailed Echelon-10 (ev3 Endovascular, Plymouth, MN, USA) microcatheter. Ten coils were used to occlude the aneurysm (for framing, Axium 3D detachable coils 4 mm × 12 cm (ev3, Covidien, Irvine, California, USA); For subsequent filling and finishing, Axium 3D detachable coils 2 mm × 6 cm, 3 mm × 8 cm, 3 mm × 6 cm, 3 mm × 8 cm, and 2 mm × 4 cm; Axium Helical detachable coils 2 mm × 8 cm, and 2 mm × 8 cm (ev3, Covidien, Irvine, California, USA); and MicroPlex 10 HyperSoft helical coils 4 mm × 10 cm and 2 mm × 6 cm (MicroVention, Tustin, California, USA)) (Fig. 2d). After the procedure, the patient recovered from general anesthesia smoothly without any neurological deficit. The patient was treated with dual antiplatelet therapy which consisted of aspirin (100 mg) and clopidogrel (75 mg). Clopidogrel was discontinued at latest follow-up (6 month), and the aspirin (100 mg) was continued for life. DSA follow-up on 6 months showed complete obliteration of the aneurysm, and the patient was symptom free (mRS 0) (Fig. 2e, f).

## Discussion

Here, we reported the successful obliteration of a ruptured and recurrent mid-basilar trunk aneurysm which failed to achieve complete occlusion by coiling or stent-assisted coiling. The treatment was completed by implanting a PED in a previous Enterprise stent.

Even though it has been proved that PED is safe and efficient in the treatment of previously coiled [7] or clipped [8] aneurysms, to apply pipeline in the setting of recurrent stented aneurysms remains the most debated area of flow diversion [6, 9]. By far, Daou et al. has reported the largest series of previously stented aneurysms treated with PED [6]. Of the 21 included cases, Enterprise was used in 52% patients and Neuroform in 48% patients. Complete aneurysm occlusion only achieved in 55.6% patients. When compared with patients with pipeline as the stand-alone treatment, 80.4% patients achieved complete occlusion. It seemed that the preexisted stent significantly undermined the hemodynamic effect of the flow diverter and pipeline should not be used in this scenario. Even though the study reported procedural events related to implanting PED in previous stent, it did not provide an approach to avoid these technical difficulties.

Despite all those dismal results, we still treated the patient with a PED. The rationale was based on three reasons. First, it would not be reasonable to repeat the already failed procedures for this refractory aneurysm which recanalized for twice. Second, the aneurysm was fusiform shape, and our experience was that PED has a very high occlusion rate for this type of aneurysm. Third, the new flow diverter FRED is a dual-layer device, with a low-porosity inner mesh and a high-porosity outer stent. Initial experience with short-term follow-up results are promising, with 80% of the treated aneurysms achieved complete occlusion at 4–6 months and 100% at 7–12 months. In summary, if we deploy the PED in a way that mimics the double layer FRED, technical events might be lowered.

In previous series, it was required to deploy the PED distal to the stent [9], and this deployment manner put the PED at high risk of twisting or flatting in the following “pull and push” maneuver since the distal end of the PED may anchor the previously placed stent strut. Besides that, if the proximal end of PED exceeds the proximal marker of the previous stent, there would be a gap between the PED and vessel wall which interfered by the previous stent strut. This gap will finally lead to mal-apposition. So, the optimal way to deploy the PED is to place it within the whole length of the previous stent, resembling the FRED flow diverter.

This deployment modality has an additional benefit to lower post procedural ischemia of brain stem, which was quietly frequently noted both in single-layer or multi-layers’ flow-diverted cases [10]. Applying this technique, only one PED was used and the perforators were directly covered by the high-porous stent instead of a PED, so the post procedure ischemia rate might be of minimal concern; this might be the account for why our patient recovered from the procedure without any neurologic deficits.

## Conclusions

We demonstrated a successful treatment of a previously stented basilar trunk aneurysm with our unique way of deploying PED in a stent. We believe that the technique may improve the performance of PED in previously stented aneurysms and may be beneficial in lowering the ischemic rate after the deployment of PED in the basilar artery. However, future studies with larger group of patients are needed to further test the safety and efficacy of this technique.

## Abbreviations

DSA: Digital subtraction angiography
mRS: Modified Rankin Scale
PED: Pipeline embolization device
SAC: Stent-assisted coiling
SAH: Subarachnoid hemorrhage
